# Serum 24,25-dihydroxyvitamin D level in general Korean population and its relationship with other vitamin D biomarkers

**DOI:** 10.1371/journal.pone.0246541

**Published:** 2021-02-19

**Authors:** Hyun-Ki Kim, Hye Jin Chung, Hương Giang Lê, Byoung-Kuk Na, Min-Chul Cho

**Affiliations:** 1 Department of Laboratory Medicine, University of Ulsan College of Medicine, Ulsan University Hospital, Ulsan, Republic of Korea; 2 College of Pharmacy and Research institute of Pharmaceutical Sciences, Gyeongsang National University, Jinju, Republic of Korea; 3 Department of Parasitology and Tropical Medicine, Gyeongsang National University College of Medicine, Jinju, Republic of Korea; 4 BK21Plus Team for Anti-aging Biotechnology and Industry, Department of Convergence Medical Science, Gyeongsang National University, Jinju, Republic of Korea; 5 Department of Laboratory Medicine, Gyeongsang National University Hospital, Gyeongsang National University College of Medicine, Jinju, Republic of Korea; 6 Institute of Health Science, Gyeongsang National University, Jinju, Republic of Korea; University of Massachusetts Medical School, UNITED STATES

## Abstract

**Background:**

Vitamin D status is presently assessed by measuring total serum concentration of 25-hydroxyvitamin D [25(OH)D]. However, 25(OH)D concentration alone might not accurately reflect vitamin D status owing to its weak relationship with various clinical indices and inconsistency across races. Recently, 24,25-dihydroxyvitamin D [24,25(OH)_2_D] and vitamin D metabolite ratio [VMR; ratio of 24,25(OH)_2_D to 25(OH)D] have emerged as vitamin D biomarkers. The present study aimed to determine the values of 24,25(OH)_2_D and VMR in healthy Koreans and compare them with other vitamin D biomarkers, including 25(OH)D and bioavailable 25(OH)D.

**Methods:**

Serum samples and medical information were collected from 200 individuals (100 females and 100 males) who underwent general health checks without self-reported symptoms. We measured 24,25(OH)_2_D concentration using liquid chromatography–tandem mass spectrometry, and concentrations of 25(OH)D and vitamin D binding protein using immunoassays. VMR and bioavailable 25(OH)D concentration were calculated using the above data. Serum parathyroid hormone level, and bone mineral density (BMD) data were collected as clinical outcomes, and the effects of the vitamin D markers on them were tested using multiple linear regression models.

**Results:**

The mean values of 25(OH)D, 24,25(OH)_2_D, VMR, and bioavailable 25(OH)D were 24.3 ± 8.5 ng/mL, 1.9 ± 1.1 ng/mL, 7.6 ± 2.5, and 3.2 ± 1.2 ng/mL, respectively. The concentration of 25(OH)D closely correlated with 24,25(OH)_2_D (R = 0.868, *P* < 0.001) and bioavailable 25(OH)D (R = 0.862, *P* < 0.001). No significant effects of 24,25(OH)_2_D, VMR, and bioavailable 25(OH)D were observed on the prediction of PTH and BMD in the multiple linear regression models.

**Conclusion:**

Our study presents the distribution of 24,25(OH)_2_D concentration and VMR in Korean population for the first time. Overall, our data reaffirm that 25(OH)D is the primary marker for determining vitamin D status in the general population.

## Introduction

Vitamin D is made or absorbed in the human body through two different pathways. The main pathway is synthesis in the skin by sunlight exposure. And the other one is absorption through food intake. The two main steps for obtaining biologically active vitamin D include 25-hydroxylation and 1α-hydroxylation. The 25-hydroxylation step occurs in the liver to produce 25-hydroxyvitamin D [25(OH)D], which is transported to the kidneys where it is converted to the active form of vitamin D, 1α,25-dihydroxyvitamin D [1α,25(OH)_2_D] [1, 2].

Vitamin D primarily plays an essential role in calcium homeostasis and development and maintenance of the skeleton. In addition, vitamin D is a multipotent vitamin that performs hormone-like functions, including endocrine functions, regulation of cell replication, and immune modulation, in various cells, tissues, and organs [3, 4]. In recent years, as numerous studies have reported the clinical significance of vitamin D, accurate assessment of vitamin D status has gained importance.

Generally, the vitamin D status of an individual is assessed by measuring the total serum concentration of 25(OH)D. The common criteria for assessment of vitamin D status include vitamin D deficiency (< 20 ng/mL) and vitamin D insufficiency (20–30 ng/mL) [2, 5, 6]. However, recent studies suggest that 25(OH)D alone may not accurately reflect the vitamin D status [7–10], though studies evaluating the relationship of 25(OH)D with bone density and fractures have had mixed findings [7–9]. Several studies have been conducted to identify indicators that accurately reflect vitamin D status rather than serum 25(OH)D level, and determination of bioavailable 25(OH)D or 24,25-dihydroxyvitamin D [24,25(OH)_2_D] levels has been suggested as a potential alternative.

Bioavailable 25(OH)D is the fraction of 25(OH)D that is not bound to vitamin D binding protein (VDBP; free and albumin-bound), and some studies have reported a stronger correlation of laboratory parameters such as serum calcium concentration, parathyroid hormone (PTH) level, and clinical parameters like bone mineral density (BMD), and vascular outcomes with bioavailable 25(OH)D concentration than that with total 25(OH)D concentration, suggesting the significance of bioavailable 25(OH)D [11–13]. The concentration of VDBP can change depending on various clinical conditions. For instance, VDBP is increased significantly in pregnancy due to elevated estrogen levels, whereas it is decreased in severe hepatic disease [14–17]. The *GC* gene that encodes VDBP exhibits about 100 polymorphisms. Two single nucleotide polymorphisms, rs7041 and rs4588, generate three isoforms of VDBP: Gc1f, Gc1s, and Gc2 [18, 19]. It has been reported that the affinity of VDBP for 25(OH)D depends on the polymorphic isoform, therefore, the *GC* genotype may also play a role in determining the concentration of bioavailable 25(OH)D [16, 17, 19]. However, in our previous study, we found that the *GC* genotype did not significantly affect the concentration of bioavailable 25(OH)D in the Korean population [20].

Studies have suggested that serum 24,25(OH)_2_D level is also a good indicator of vitamin D status [10, 21]. 24,25(OH)_2_D is the main product of 25(OH)D catabolism, and the enzymatic synthesis of 24,25(OH)_2_D is directly proportional to the concentration of 25(OH)D, thus the concentration of the two metabolites is strongly correlated with circulation [22]. In addition, the expression of the enzyme 24-hydroxylase (CYP24A1), which converts 25(OH)D to 24,25(OH)_2_D, is partially regulated by vitamin D receptor activity [23, 24]. Since the production of 24,25(OH)_2_D is dependent on 25(OH)D concentration and the regulated expression of the vitamin D receptor in CYP24A1, therefore, 24,25(OH)_2_D concentration could reflect 25(OH)D concentration [25].

In addition, recent findings suggest that adequacy of vitamin D may be reflected by the ratio of serum 24,25(OH)_2_D concentration to serum 25(OH)D concentration, known as the vitamin D metabolite ratio (VMR) [10, 21]. VMR relies primarily on CYP24A1 expression, which is downregulated in vitamin D deficiency; therefore, VMR is also expected to reduce in vitamin D deficiency. A previous study has shown that the VMR values between whites and blacks in a randomized community cohort were similar, but blacks often had low 25(OH)D concentrations without any signs of vitamin D deficiency, indicating that VMR could be a potential vitamin D biomarker in blacks instead of 25(OH)D [25].

It has been reported that vitamin D-related metabolism varies according to race, and research has been conducted to assess the potential of bioavailable 25(OH)D and 25(OH)D as biomarkers of vitamin D status in general Korean population [20, 26]. However, research related to 24,25(OH)_2_D and VMR has not yet been conducted in Asians, including Koreans. Furthermore, no studies have compared various vitamin D markers, including 25(OH)D, 24,25(OH)_2_D, VMR, and bioavailable vitamin D, in the same subjects simultaneously. Therefore, in this study, we determined the values of 24,25(OH)_2_D and VMR in healthy Koreans and compared them with other vitamin D biomarkers, including 25(OH)D and bioavailable vitamin D.

## Materials and methods

### Study subjects

A total of 200 healthy individuals who underwent general medical check-ups without any self-reported symptoms in Gyeongsang National University hospital from July 2019 to October 2019 were enrolled in this prospective study. The study subjects included 100 males and 100 females. There were no selection criteria or exclusion criteria for the study subjects. All patients who had undergone medical examination during a specific period were enrolled. 100 patients each, male and female, were included in the study.

Demographic and laboratory data, including age, sex, and serum albumin, calcium, phosphate, and PTH concentrations, were collected from electronic medical records. For female subjects, menopause and menopause age were surveyed, and BMD data, measured using dual energy x-ray absorptiometry (DEXA), were collected if available. At the time of enrollment, blood samples of the subjects were collected, and serum and leukocytes were separated and stored at −80 °C. The study protocol was approved by the Institutional Review Board of Gyeongsang National University Hospital (approval number: 2019-08-008). Written informed consent was obtained from all participants.

### Vitamin D measurements

Total 25(OH)D concentration was measured using Elecsys vitamin D total electrochemiluminescence binding assay (Roche Diagnostics, Mannheim, Germany) and Cobas 8000 e602 analyzer (Roche Diagnostics).

Total 24,25(OH)_2_D_3_ concentration was measured using solid-phase extraction and subsequent liquid chromatography–tandem mass spectrometry (LC-MS/MS), as described by van den Ouweland et al. [27] with certain modifications. Briefly, an internal standard and stable isotope-labeled d6-24,25(OH)_2_D_3_ were added to 200 μL of serum sample. Next, methanol was added, and the solution was vortex-mixed and incubated at 4 °C for 10 min for protein precipitation. The reaction mixture was centrifuged at 4 °C, 12 000 *g* for 10 min; the supernatant was mixed with phosphate-buffered saline and loaded onto a solid-phase extraction cartridge. After performing solid-phase extraction, the eluted fraction was evaporated under vacuum. The dried residue was reconstituted in 75% methanol, and 5 μL was injected into the LC-MS/MS system for analysis. The LC-MS/MS system consisted of an Agilent 1260 high-performance liquid chromatography (HPLC) system (Agilent, Germany) and an Agilent 6460 triple quadrupole mass spectrometer (Agilent, Singapore) equipped with an electrospray ionization source. Kinetex^®^ Biphenyl column (particle size, 2.6 μm; i.d., 3.0 mm; length, 100 mm) (Phenomenex, Torrance, CA, USA) was used for HPLC separation. The HPLC mobile phase consisted of 0.1% aqueous formic acid and methanol, and a gradient program was used at a flow rate of 0.4 mL/min. Electrospray ionization was performed in the positive mode with nitrogen as the nebulizer, turbo spray, and curtain gas. The multiple reaction monitoring detection method was used for detection of the analytes. The following transitions were monitored: *m/z* 417 → 381 for 24,25(OH)_2_D_3_, and *m/z* 423 → 387 for d6-24,25(OH)_2_D_3_. The concentration of 24,25(OH)_2_D_3_ in the serum samples was determined from a calibration curve of the peak area ratio of the analyte to the internal standard. The calibration curves were linear over the ranges studied, with *r*^2^ > 0.999. The limit of quantitation of 24,25(OH)_2_D_3_ was 0.2 ng/mL. The accuracy ranged from 90.7% to 104.0%, and the coefficients of variation of the assay (intra-batch and inter-batch precisions) were less than 11.6%.

### Other measurements

VDBP concentration was measured using an enzyme-linked immunosorbent assay kit (R&D Systems, Minneapolis, MN, USA) according to the manufacturer’s protocol. Estimated glomerular filtration rate (eGFR) was calculated using the Chronic Kidney Disease Epidemiology Collaboration (CKD-EPI) creatinine equation [28].

### Calculation of VMR and bioavailable 25(OH)D concentration

VMR was calculated by dividing serum 24,25(OH)_2_D concentration by serum 25(OH)D concentration and then multiplying by 100 [25]. Based on total 25(OH)D, VDBP, and albumin concentrations, bioavailable 25(OH)D concentration was calculated using the equations from previous studies [20, 29]. According to our previous research [20], genotype-independent VDBP binding affinity (0.7 × 10^9^ M^−1^) was used for calculation of bioavailable 25(OH)D concentration.

### Statistical analysis

At the study design, sample size for the linear regression model between PTH and 24,25(OH)_2_D with 25(OH)D covariate was calculated using a simple formula which is proposed by Hsieh [30]. When α = 0.05 and β = 0.1 (meaning power = 90%), assumed the correlation coefficient between PTH and 24,25(OH)_2_D was 0.25, and the correlation coefficient between 24,25(OH)_2_D and 25(OH)D was 0.4 based on the previous study data [25], the required total sample size was 195.3 (Actually we recruited 200 subjects).

Result data are presented as mean ± standard deviation, and were compared between sexes using t-tests, except for PTH. As PTH was non-normally distributed, PTH data are presented as median and interquartile range, and compared using Mann-Whitney U test between sexes. For examination of bivariate relationships between parameters, Pearson’s correlation (R), linear regression, and scatterplots were used. For determination of correlation between PTH with vitamin D status markers, Spearman’s rank correlation coefficients (R_s_) were calculated. Multiple linear regression model was used to test the association of age (categorical; 18–34, 35–49, 50–64, 65–78 years), sex, and eGFR (categorical; ≥ 90 and < 90 mL/min/1.73 m^2^) with 24,25(OH)_2_D and 25(OH)D in order to estimate how they modified the relationship between 24,25(OH)_2_D and 25(OH)D. Multiple linear regression models on log-transformed PTH adjusted for 25(OH)D were used to investigate the influence of other vitamin D status markers, including interaction terms of the other vitamin D status markers with 25(OH)D, on the prediction of PTH. Multiple linear regression models on T-score adjusted for 25(OH)D, age (continuous, years), and menopausal status were used to investigate the influence of other vitamin D status markers, including interaction terms of the other vitamin D status markers with 25(OH)D, on the prediction of T-score. The two-sided significance level was set to 0.05. All statistical analyses were performed using R version 3.6.3. (R Foundation for Statistical Computing, Vienna, Austria).

## Results

### Subject characteristics and distribution of vitamin D status markers according to sex and age

We analyzed samples from 200 subjects, including 100 females and 100 males. Baseline characteristics, including age, calcium, VDBP, and VMR, were not significantly different among the subjects. PTH and phosphorus levels were higher in females than those in males. Albumin, 25(OH)D, 24,25(OH)_2_D, and bioavailable 25(OH)D levels were lower in females than those in males (Table 1). Concentrations of 25(OH)D (R = 0.381, *P* < 0.001), 24,25(OH)_2_D (R = 0.167, *P* = 0.018), and bioavailable 25(OH)D (R = 0.265, *P* = 0.001) positively correlated with age, while VMR (R = –0.079, *P* = 0.267) did not exhibit a significant correlation with age. Elderly females (65–78 years) displayed the lowest median VMR value (Table 2).

**Table 1:** Subject characteristics and vitamin D parameters.

Parameters	Total (n = 200)	Female (n = 100)	Male (n = 100)	*P*-value*
**Age, year**	48.7 (13.5)	50.2 (13.8)	47.2 (12.9)	0.105
**Albumin, g/dL**	4.61 (0.27)	4.57 (0.23)	4.66 (0.29)	0.027
**Calcium, mg/dL**	9.36 (0.34)	9.36 (0.34)	9.37 (0.33)	0.818
**Phosphorus, mg/dL**	3.36 (0.50)	3.56 (0.47)	3.17 (0.45)	< 0.001
**eGFR, mL/min/1.73 m**^**2**^	99.87 (14.55)	102.53 (15.33)	97.20 (13.27)	0.009
**PTH, pg/mL**	32.93 (14.65)	36.18 (15.43)	29.98 (14.99)	0.003
**VDBP, μg/mL**	238.56 (54.02)	240.62 (56.12)	236.50 (52.03)	0.658
**25(OH)D, ng/mL**	24.27 (8.53)	22.31 (7.14)	26.22 (9.35)	0.001
**24,25(OH)**_**2**_**D, ng/mL**	1.93 (1.13)	1.70 (0.99)	2.17 (1.22)	0.003
**VMR**	7.63 (2.46)	7.31 (2.66)	7.95 (2.21)	0.069
**Bioavailable 25(OH)D, ng/mL**	3.17 (1.15)	2.88 (0.98)	3.47 (1.24)	0.002

Values are presented as mean (standard deviation), except for PTH [median (interquartile range)].

*P-values are given by Mann-Whitney U test for PTH, and by t-tests for other parameters.

eGFR: expected glomerular filtration rate; PTH: parathyroid hormone; VDBP: vitamin D binding protein; 25(OH)D: 25-hyroxyvitamin D; 24,25(OH)_2_D: 24,25-dihydroxyvitamin D; VMR: vitamin D metabolite ratio

**Table 2:** Distribution of vitamin D biomarkers according to sex and age, excluding seven subjects with PTH > 65 pg/mL.

Age group (years) / sex	No. of subjects	Median values of Vitamin D status markers (IQR)			
		25(OH)D, ng/mL	24,25(OH)_2_D, ng/mL	VMR	bioavailable 25(OH)D, ng/mL
**18–34**					
**Total**	36	19.10 (6.65)	1.41 (0.97)	7.67 (3.07)	2.57 (0.85)
**Female**	14	16.60 (2.10)	1.34 (0.43)	8.53 (3.86)	2.32 (0.75)
**Male**	22	21.10 (11.73)	1.42 (1.01)	7.52 (2.47)	2.97 (0.93)
**35–49**					
**Total**	61	21.00 (7.60)	1.66 (1.28)	7.94 (4.49)	2.65 (1.26)
**Female**	32	19.15 (7.28)	1.14 (0.94)	6.24 (3.58)	2.26 (0.89)
**Male**	29	23.40 (8.80)	2.01 (1.22)	8.16 (4.64)	3.27 (1.11)
**50–64**					
**Total**	74	26.25 (9.95)	1.89 (1.26)	7.43 (2.67)	3.32 (1.34)
**Female**	36	24.50 (6.00)	1.65 (1.15)	7.08 (3.38)	2.93 (0.99)
**Male**	38	26.40 (10.70)	2.03 (1.23)	7.61 (2.70)	3.72 (1.28)
**65–78**					
**Total**	22	26.60 (7.23)	1.86 (1.05)	7.19 (2.93)	3.36 (1.46)
**Female**	13	27.20 (6.90)	1.78 (0.81)	5.92 (2.83)	3.56 (1.47)
**Male**	9	26.40 (6.60)	2.09 (1.31)	7.80 (2.76)	3.27 (1.41)

PTH: parathyroid hormone; IQR: interquartile range; 25(OH)D: 25-hyroxyvitamin D; 24,25(OH)_2_D: 24,25-dihydroxyvitamin D; VMR: vitamin D metabolite ration

### Relationship of 25(OH)D with 24,25(OH)_2_D and VMR

**Fig 1:**
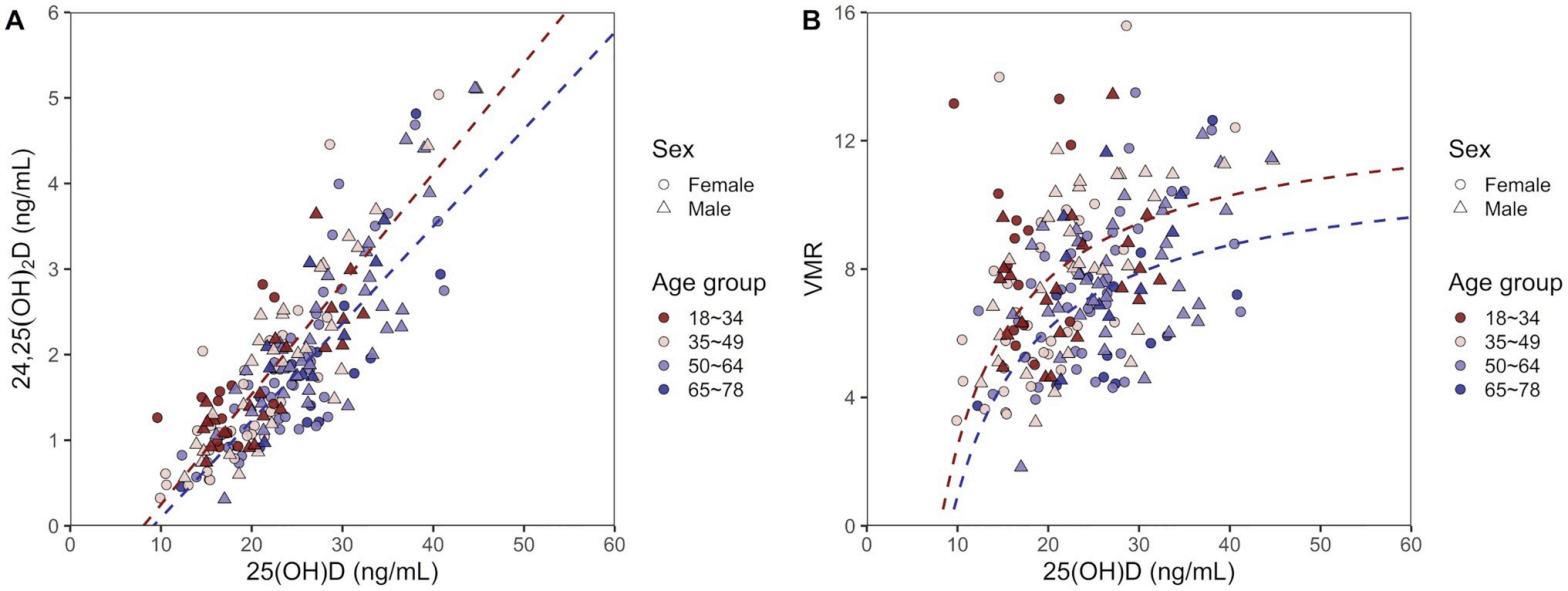
Relationship of 25-hydroxy vitamin D with 24,25-dihydroxyvitamin D and vitamin D metabolite ratio. (A) Association between 25(OH)D and 24,25(OH)_2_D. Circles represent females, and triangles represent males; different colors represent different age groups. Dashed lines represent linear fit generated using ordinary linear regression with the slopes adjusted for subjects in the age group 18–34 years (red) or 65–78 years (blue). Linear regression was performed using all subjects, but subjects with 25(OH)D above 60 ng/mL were truncated for presentation. (B) Association between 25(OH)D and VMR. Circles represent females, and triangles represent males; different colors represent different age groups. Dashed lines indicate VMR values calculated from the corresponding 24,25(OH)_2_D and 25(OH)D concentrations according to the linear regression between 25(OH)D and 24,25(OH)_2_D. 25(OH)D: 25-hyroxyvitamin D; 24,25(OH)_2_D: 24,25-dihydroxyvitamin D; VMR: vitamin D metabolite ratio.

Concentration of 25(OH)D strongly correlated with the concentration of 24,25(OH)_2_D (R = 0.868, *P* < 0.001; Fig 1A). Linear regression analysis indicated that [24,25(OH)_2_D] = 0.129 × [25(OH)D] − 1.040 for subjects in the age group 18–34 years. The slope for subjects in the age groups 50–64 years (slope = 0.118, *P* = 0.034) and 65–78 years (slope = 0.113, *P* = 0.012) was significantly different from that for subjects in the age group 18–34 years. The slope was also significantly different between subjects with eGFR ≥ 90 mL/min/1.73 m^2^ and subjects with eGFR < 90 mL/min/1.73 m^2^ (*P* = 0.006). The slope was not significantly different between the two sexes (*P* = 0.580). The expected value of VMR [24,25(OH)_2_D/25(OH)D × 100] was calculated from the concentration of 24,25(OH)_2_D corresponding to the concentration of 25(OH)D using the linear regression equations between them: VMR = (−1.040 × [25(OH)D] + 0.129) × 100 for subjects in the age group 18–34 years, and VMR = (−1.040 × [25(OH)D] + 0.113) × 100 for subjects in the age group 65–78 years (Fig 1B). VMR exhibited a positive correlation with 25(OH)D concentration (R_s_ = 0.405, *P* < 0.001).

### Relationship of bioavailable 25(OH)D with 25(OH)D, 24,25(OH)_2_D, and VMR

**Fig 2:**
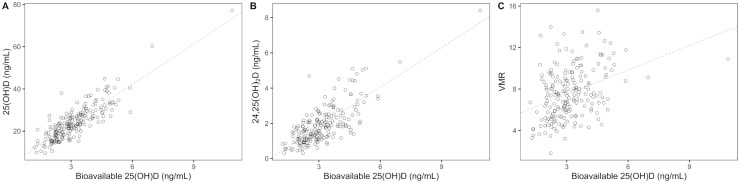
Relationship of bioavailable 25-hydroxyvitamin D with 25-hydroxyvitamin D, 24,25-dihydroxyvitamin D, and vitamin D metabolite ratio. (A) Association between bioavailable 25(OH)D and 25(OH)D. (B) Association between bioavailable 25(OH)D and 24,25(OH)_2_D. (C) Association between bioavailable 25(OH)D and VMR. Dashed lines represent linear fit generated using ordinary linear regression. 25(OH)D: 25-hyroxyvitamin D; 24,25(OH)_2_D: 24,25-dihydroxyvitamin D; VMR: vitamin D metabolite ratio.

Bioavailable 25(OH)D concentration correlated strongly with the concentrations of 25(OH)D (R = 0.862, *P* < 0.001) and 24,25(OH)_2_D (R = 0.765, *P* < 0.001), and weakly with VMR (R = 0.367, *P* < 0.001) (Fig 2). Concentration of VDBP positively correlated with the concentration of 25(OH)D (R = 0.165, *P* = 0.020), while it had no significant correlation with 24,25(OH)_2_D concentration (R = 0.131, *P* = 0.064) and VMR (R = 0.058, *P* = 0.414).

### Relationship of vitamin D status markers with PTH and bone marrow density

**Fig 3:**
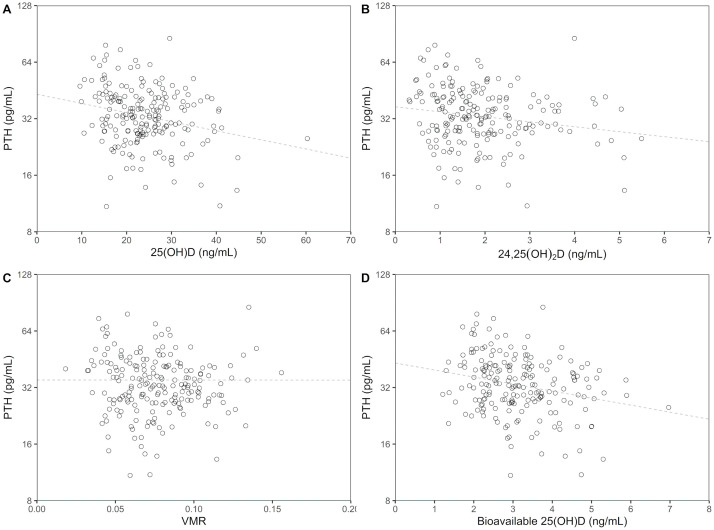
Relationship of parathyroid hormone with vitamin D parameters. Association of PTH with (A) 25(OH)D, (B) 24,25(OH)_2_D, (C) VMR, and (D) bioavailable 25(OH)D. PTH was log-transformed. Dashed lines represent linear fit generated using ordinary linear regression. PTH: parathyroid hormone; 25(OH)D: 25-hyroxyvitamin D; 24,25(OH)_2_D: 24,25-dihydroxyvitamin D; VMR: vitamin D metabolite ratio.

PTH concentration negatively correlated with the concentrations of 25(OH)D (R_s_ = −0.222, *P* = 0.002), 24,25(OH)_2_D (R_s_ = −0.191, *P* = 0.007), and bioavailable 25(OH)D (R_s_ = −0.255, *P* < 0.001) to a similar degree. Correlation between PTH concentration and VMR (R_s_ = −0.092, *P* = 0.193) was less significant (Fig 3). In linear regression models with log-transformed PTH as the response variable, no significant differences between the model with 25(OH)D as the only predictor and the models with 24,25(OH)_2_D (*P* = 0.492), bioavailable 25(OH)D (*P* = 0.298), or VMR (*P* = 0.385) as predictors including the interaction terms with 25(OH)D were observed, indicating that these markers did not have a significant effect on prediction of PTH concentration.

Dual energy x-ray absorptiometry test results were available for 88 females. In multiple linear regression models for predicting T-score with age and menopause status as covariates, 25(OH)D (*P* = 0.396), 24,25(OH)_2_D (*P* = 0.795), bioavailable 25(OH)D (*P* = 0.373), and VMR (*P* = 0.981) did not have significant effects.

## Discussion

In this study, we present the distribution of 25(OH)D, bioavailable 25(OH)D, VDBP, 24,25(OH)_2_D, and VMR among Koreans based on demographic characteristics. In addition, we demonstrated the correlation among vitamin D status markers. To our knowledge, this is the first report presenting serum 24,25(OH)_2_D concentration and VMR in general Korean population. We also attempted to investigate the influence of vitamin D status markers on the prediction of PTH or BMD T-score.

Concentration of 25(OH)D was lower in young adults and female subjects than that in other subjects. This tendency was also observed in a previous study based on the Korea National Health and Nutrition Examination Survey data representing the total non-institutionalized civilian population of Korea [31], and in reports from China and Thailand [32, 33]. It is surmised that this phenomenon might be a result of influence of environmental factors, including indoor lifestyle and sun avoidance.

Similar to 25(OH)D concentration, the concentration of 24,25(OH)_2_D was lower in young adults and female subjects than that in other subjects. Several studies have reported that VMR is low in individuals with low serum 25(OH)D [34, 35]. Our results are consistent with those of previous studies that demonstrate overall positive correlation between 25(OH)D and VMR. Interestingly, VMR was paradoxically lower among elderly females (65–78 years). This phenomenon can be attributed to renal function. Lower eGFR is associated with lower circulating 24,25(OH)_2_D, whereas no correlation has been observed between 25(OH)D and eGFR [10, 22]; therefore, VMR decreases with decreasing eGFR. As renal function generally decreases with aging [36], elderly subjects with decreased renal function might have lower VMR irrespective of higher 25(OH)D concentration. Similarly, in our study, the increment in 24,25(OH)_2_D per unit higher 25(OH)D (slope) was reduced in subjects with eGFR < 90 mL/min/1.73 m^2^. However, the mechanism underlying the relationship between serum 24,25(OH)_2_D concentration and eGFR is unclear, and it could involve other factors, including age and sex. Further studies are warranted to reveal the relationship between VMR and age.

A strong correlation was observed between 25(OH)D and 24,25(OH)_2_D, and it is to be expected considering that there is direct enzymatic conversion of 25(OH)D into 24,25(OH)_2_D. VMR moderately correlated with 25(OH)D, as expected from the linear relationship between 25(OH)D and 24,25(OH)_2_D. These correlations have also been shown by other studies [25, 35, 37]. In our study, a positive correlation was observed between 25(OH)D and VDBP (R = 0.165, *P* = 0.020), whereas no significant correlation was observed between VMR and VDBP (R = 0.058, *P* = 0.414). VDBP concentration influences the concentration of 25(OH)D, independent of vitamin D sufficiency, but does not influence VMR [38]. A previous study reported that VMR values were equivalent between blacks and whites, while serum concentrations of 25(OH)D and 24,25(OH)_2_D were significantly lower in blacks than those in whites [25]. The *Gc1f* allele frequency of general Korean population is reported to be 42–46% [26, 39], which is related to low VDBP and 25(OH)D concentrations [29]. Therefore, VMR, which seems to be less influenced by VDBP than 25(OH)D, may be advantageous as a biomarker for determining the vitamin D status of Koreans.

In our study, no significant effects of 24,25(OH)_2_D, VMR, and bioavailable 25(OH)D were observed on the prediction of PTH and BMD in the multiple linear regression models. However, a previous study showed that, in a cohort of 278 participants with chronic kidney disease, 24,25(OH)_2_D (R = −0.44, *P* < 0.001) showed stronger association with PTH than that with 1α,25(OH)_2_D (R = −0.16, *P* = 0.01) or 25(OH)D (R = −0.22, *P* < 0.001) [10]. Furthermore, after adjustment for several factors, including 1α,25(OH)_2_D and 25(OH)D, each 1 ng/mL lower 24,25(OH)2D was associated with an estimated 13% higher geometric mean PTH. The negative result observed in our study might be due to the small size of study population, narrow range of PTH level, or missing confounding factors, which resulted in reduced statistical power. Currently, there are insufficient clinical studies on 24,25(OH)_2_D and VMR. Further studies are warranted to reveal the advantages of 24,25(OH)_2_D and VMR as vitamin D status markers.

The present study had two main limitations. First, we collected samples only from individuals who underwent general medical check-ups without self-reported symptoms, and there were no specific criteria or questionnaire for the study. Therefore, the study population might not be representative of the general healthy Korean population. Second, environmental factors that could affect vitamin D concentration, including food, outdoor activity period, use of sunscreen, and vitamin D supplement intake, were not surveyed. Due to these limitations, it may not be possible to eliminate all confounding factors in the analysis of various vitamin D status biomarkers. However, this is the first report on vitamin D status markers for Koreans; our study provides rough estimates of vitamin D status markers, including 24,25(OH)_2_D, which could guide future studies.

In conclusion, our results provide a rough estimate of the distribution of 24,25(OH)_2_D and VMR in general Korean population. These results may provide background data for future studies on 24,25(OH)_2_D and other markers related to vitamin D status, especially in the Korean population. Although elderly females of our study population exhibited lower VMR despite having higher 25(OH)D concentration, and it seems to be worth investigating whether 24,25(OH)2D or VMR is advantageous in assessing vitamin D status and metabolism in such a specific group; additional benefits of 24,25(OH)_2_D or VMR over 25(OH)D as a marker for evaluation of vitamin D status in general population could not be verified in this study. Overall, our data reaffirm that 25(OH)D is the primary marker for determining vitamin D status in the general population.
